# In the Search for Biomarkers of Pulmonary Arterial Hypertension, Are Cytokines IL-2, IL-4, IL-6, IL-10, and IFN-Gamma the Right Indicators to Use?

**DOI:** 10.3390/ijms241813694

**Published:** 2023-09-05

**Authors:** Michał Tomaszewski, Paulina Mertowska, Martyna Janczewska, Agnieszka Styczeń, Sebastian Mertowski, Kamil Jonas, Ewelina Grywalska, Grzegorz Kopeć

**Affiliations:** 1 Department of Cardiology, Medical University of Lublin, Jaczewskiego 8, 20-954 Lublin, Poland; michal.tomaszewski@umlub.pl (M.T.); m.janczewska2002@gmail.com (M.J.); styczen.agnieszka@gmail.com (A.S.); 2 Department of Experimental Immunology, Medical University of Lublin, Chodzki 4a, 20-093 Lublin, Poland; paulina.mertowska@umlub.pl (P.M.); ewelina.grywalska@umlub.pl (E.G.); 3 Pulmonary Circulation Centre, Department of Cardiac and Vascular Diseases, Jagiellonian University Medical College, Centre for Rare Cardiovascular Diseases, John Paul II Hospital, ul. Pradnicka 80, 31-202 Krakow, Poland; kamil.jns@gmail.com (K.J.); grzegorzkrakow1@gmail.com (G.K.)

**Keywords:** pulmonary arterial hypertension, cytokines, immune responses, inflammation

## Abstract

Pulmonary arterial hypertension (PAH) is a complex disorder characterized by increased pressure in the pulmonary arteries, leading to right heart failure. While the exact mechanisms underlying PAH are not fully understood, cytokines have been implicated in the pathogenesis of the disease. Cytokines play a crucial role in regulating immune responses and inflammation. These small proteins also play a key role in shaping the immunophenotype, which refers to the specific characteristics and functional properties of immune cells, which can have a significant impact on the development of PAH. The aim of this study was to determine the immunophenotype and the concentration of selected cytokines, IL-2, IL-4, IL-6, IL-10, and IFN-gamma, in patients diagnosed with PAH (with particular emphasis on subtypes) in relation to healthy volunteers. Based on the obtained results, we can conclude that in patients with PAH, the functioning of the immune system is deregulated as a result of a decrease in the percentage of selected subpopulations of immune cells in peripheral blood and changes in the concentration of tested cytokines in relation to healthy volunteers. In addition, a detailed analysis showed that there are statistically significant differences between the PAH subtypes and the tested immunological parameters. This may indicate a significant role of the immune system in the pathogenesis of PAH.

## 1. Introduction

**Figure 1:**
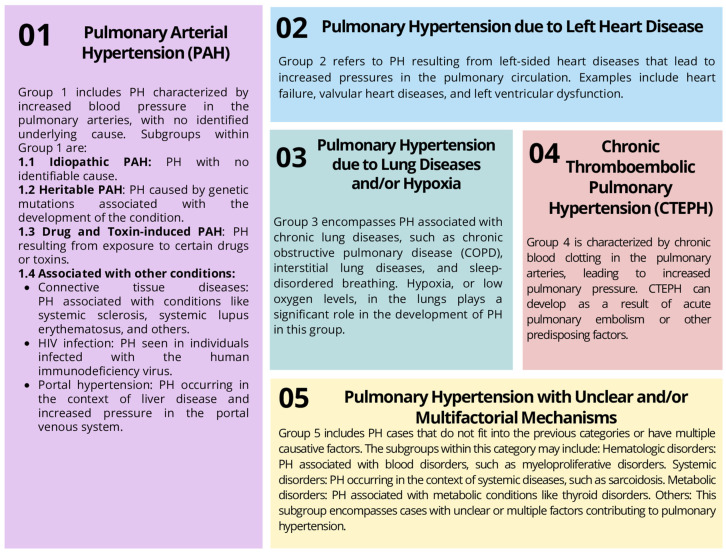
Pulmonary hypertension classification (based on [2]).

Pulmonary hypertension (PH) is a pathological increase in pressure in the pulmonary artery which can develop as a result of various underlying diseases of the heart, lungs, or pulmonary vessels [1]. The diagnosis of PH requires invasive hemodynamic measurements that allow for an effective diagnosis, but it should be emphasized that this disease is very diverse, varying in etiology, clinical picture, pathophysiology, hemodynamic characteristics, and treatment strategies. Therefore, PH was divided into five clinical classes [2] (Figure 1).

Despite advances in treatment, PH continues to have high rates of morbidity and mortality, possibly due to delayed diagnosis. To ascertain the presence of PH, dedicated medical institutions employ echocardiography, meticulously analyzing parameters such as tricuspid valve velocity and the morphological dimensions of the right ventricle. These parameters facilitate the stratification of the disease likelihood into discrete categories: low, moderate, or high. This stratification paradigm plays a pivotal role in the diagnostic algorithm delineated by the European Society of Cardiology (ESC) [2]. Should the post-echocardiography analysis indicate a low likelihood of PH, the diagnostic algorithm advocates the exploration of alternative etiologies accounting for the patient’s clinical manifestations. Conversely, individuals categorized with moderate to high likelihood are advised to undergo a comprehensive array of diagnostic procedures, encompassing electrocardiograms (ECGs), arterial blood gas analysis, pulmonary function assessment, chest radiography, and high-resolution computed tomography. Concomitantly, the pulmonary diffusing capacity for carbon monoxide warrants evaluation. Upon exclusion of pathologies of the left cardiac chambers and pulmonary system, it becomes imperative to channel the patient to an institution specializing in the comprehensive diagnosis and therapeutic management of pulmonary hypertension. Within such institutions, right heart catheterization is typically executed [3,4]. PAH is diagnosed through invasive hemodynamic measurements to determine if mean pulmonary arterial pressure (mPAP) is ≥20 mmHg, pulmonary capillary wedge pressure (PCWP) is ≤15 mmHg, and pulmonary vascular resistance (PVR) is ≥2 WU [2]. Specific diagnostic tests can identify different types of PAH, but to effectively treat PH, it is important to identify the root cause.

**Figure 2:**
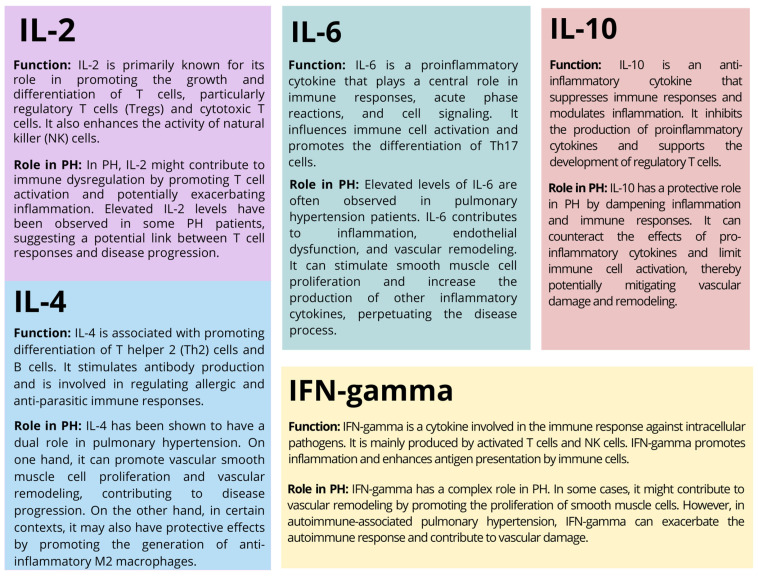
The potential roles of selected cytokines in the course of PH [5,6,7,8].

The involvement of the immune system in PH is the result of a complex interaction of immune cells, cytokines, growth factors, and pulmonary vascular cells. Chronic inflammation and activation of immune cells may contribute to remodeling of the pulmonary arteries. This remodeling includes structural changes, such as thickening of the vessel walls, narrowing of the vessel lumen, and lesion formation [5]. Immune cells release growth factors that promote the proliferation of vascular smooth muscle cells that are involved in vasoconstriction. Immune cells, especially macrophages and T cells, are found in the lung tissues and blood vessels of people with pulmonary hypertension. These cells release proinflammatory molecules called cytokines that can promote inflammation, cell proliferation, and vascular remodeling [6,7,8]. Some evidence suggests that autoimmune mechanisms may be involved in some forms of pulmonary hypertension, such as pulmonary hypertension associated with connective tissue disease. Autoimmune processes are processes in which the immune system mistakenly attacks the body’s own tissues. In such cases, autoantibodies and immune complexes may contribute to inflammation and vascular damage in the pulmonary arteries [5,6,7,8]. Understanding the role of the immune system in pulmonary hypertension has opened up new avenues for therapeutic intervention. Immunomodulatory therapies, such as targeting specific cytokines or immune cell pathways, are being explored as potential treatments to alleviate inflammation and vascular remodeling. Studying cytokines can help improve understanding of the disease, as they regulate the severity and type of inflammatory response (Figure 2) and, acting through specific receptors, can trigger cells to proliferate and secrete biologically active substances [7,8].

In our research, we focused on studying the expression of immunological markers in patients with PAH, with a particular emphasis on suppressive molecules. Our specific goals included evaluating the levels of certain cytokines (interferon-gamma, IL-2, IL-4, IL-6, and IL-10) in the plasma of all study groups, compared with a control group. Our aim was to investigate the potential role of these cytokines in the molecular pathomechanisms of PAH, and to identify any markers that could be used for diagnosing the condition.

## 2. Results

The first stage of our research was based on the basic immunophenotype of all analyzed patients, both from the study group and healthy volunteers. The obtained results are presented in Table 1.

**Figure 3:**
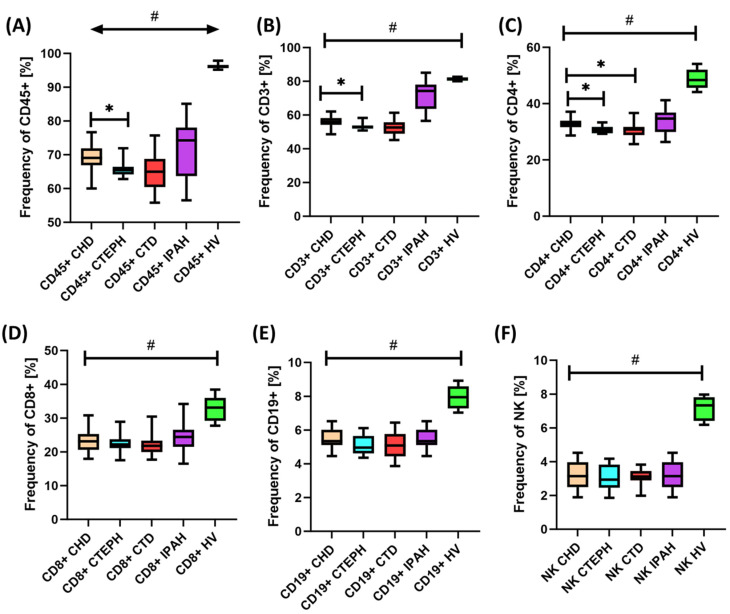
Graphic illustration of the results obtained from the analysis of peripheral blood immunophenotypes of patients with CTEPH, CHD, CTD, and IPAH in relation to healthy volunteers (**A**) Frequency of CD45+ (**B**) Frequency of CD3+ (**C**) Frequency of CD4+ (**D**) Frequency of CD8+ (**E**) Frequency of CD19+ (**F**) Frequency of NK cells. The chart shows the median, quartiles, and minimum and maximum values. HV means healthy volunteers; * indicates statistically significant results between individual groups of patients; and # indicates results are significant in relation to the results of healthy volunteers. For better perception, each disease subdivision is marked with a different color.

Based on these results, we can conclude that all the analyzed subpopulations of immune cells are significantly lower in patients with PAH than in healthy individuals. Moreover, a detailed analysis of particular PAH subtypes also showed a number of statistically significant differences in immunophenotype (Table 2). The observed immunophenotype disorders in patients with PAH also show a number of changes not only in relation to the control group (Figure 3A–F), but also between individual subtypes of the disease, which indicates a significant role of the immune system in the pathogenesis of this type of disease (Figure 3A–C).

In the next stage of the study, the concentration of cytokines IL-2, IL-4, IL-6, IL-10, and IFN-γ in the plasma of patients diagnosed with PAH as compared with healthy volunteers was analyzed. The obtained results are presented in Table 3.

**Figure 4:**
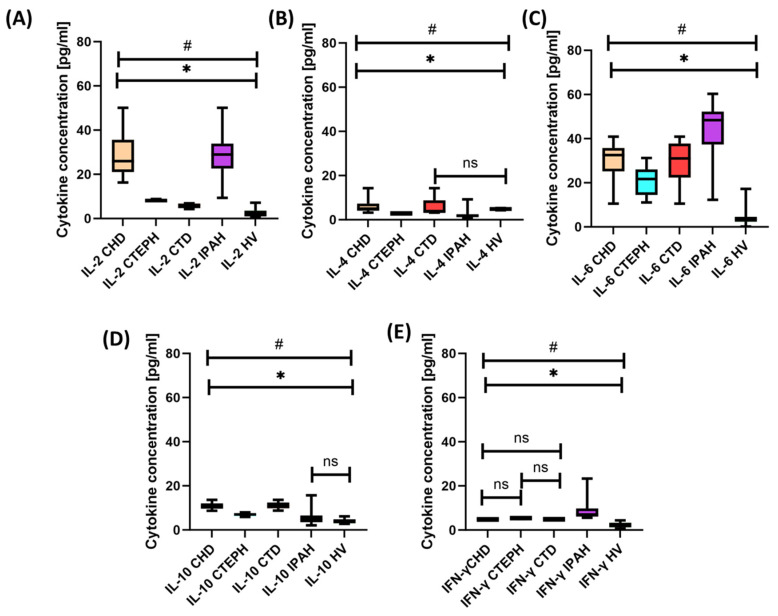
Graphic illustration of the results obtained from the analysis of cytokine concentration patients with CTEPH, CHD, CTD, and IPAH in relation to healthy volunteers. (**A**) IL-2 cytokine concentration (**B**) IL-4 cytokine concentration (**C**) IL-6 cytokine concentration (**D**) IL-10 cytokine concentration (**E**) IFN-γ cytokine concentration. The chart shows the median, quartiles, and minimum and maximum values. HV means healthy volunteers; * indicates statistically significant results between individual groups of patients; # indicates that results are significant in relation to the results of healthy volunteers; and ns means there are no statistically significant results between particular groups of patients. For better perception, each disease subdivision is marked with a different color.

As can be seen in Table 3, all cytokines tested, with the exception of IL-4, were significantly elevated in PAH patients compared with healthy volunteers. The highest difference was obtained for IL-6, whose increase compared with healthy volunteers was over 9-fold, while the lowest difference was for IFN-γ, where the recorded increase was over 2.5 times higher. A detailed analysis of the obtained results, taking into account the individual PAH subtypes, also showed a number of significant changes in the level of the tested cytokines compared with that of healthy volunteers (Supplementary Materials Table S1; Figure 4).

In the case of IL-2, the highest concentrations were achieved in patients with IPAH and CHD, which were more than 10- and 11-fold higher, respectively, than in healthy volunteers, and also significantly differed between all PAH subtypes analyzed. In the case of IL-4, a significant increase in its concentration was observed in patients with CHD (1.26 times higher), and a decrease was observed in patients with CTEPH (1.63 times) and IPAH (2.68 times) compared with healthy volunteers. The highest concentrations among all tested cytokines were recorded for IL-6, for which the increase was 14.5 times for IPAH, 9.77 times for CTD, 6.99 times for CTEPH, and 5.65 times for CHD. In the case of IL-10 and IFN-y, we observed a statistically significant increase in their concentration in all PAH subtypes compared with that in healthy individuals (Table 4). Due to such different concentration levels of the tested cytokines, we also showed significant differences between the individual PAH subunits (Table 4). This may indicate disorders in the functioning of the immune system, which may be involved in the pathogenesis and progression of PAH. There were no statistically significant correlations between the concentration of the cytokines tested (interferon-gamma, IL-2, IL-4, IL-6, and IL-10) and selected clinical and laboratory parameters in patients with different types of PAH.

**Figure 5:**
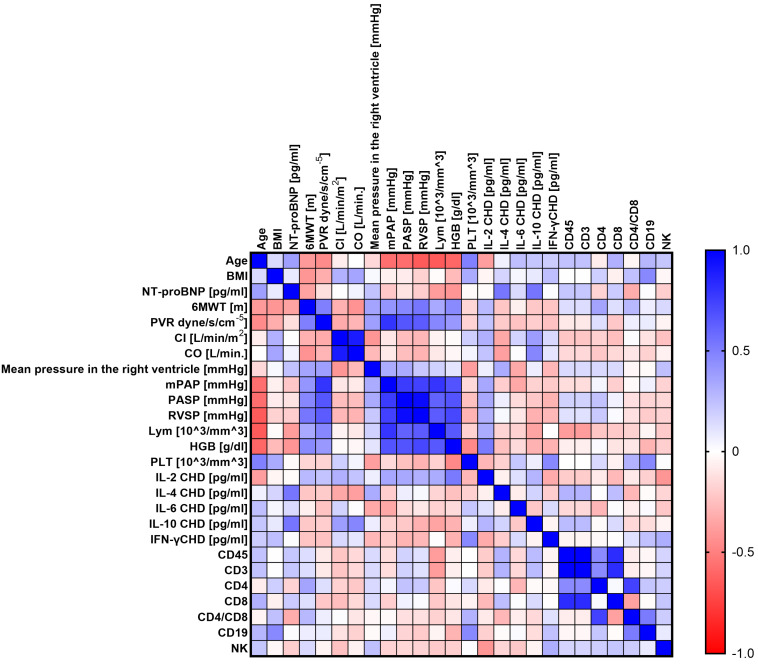
Graphical representation of Sperman’s rank correlation analysis for selected parameters of the immune system and clinical parameters in patients with CHD. Positive correlations are marked in blue and negative correlations in red. The intensity of the color is synonymous with the strength of the given correlation.

**Figure 6:**
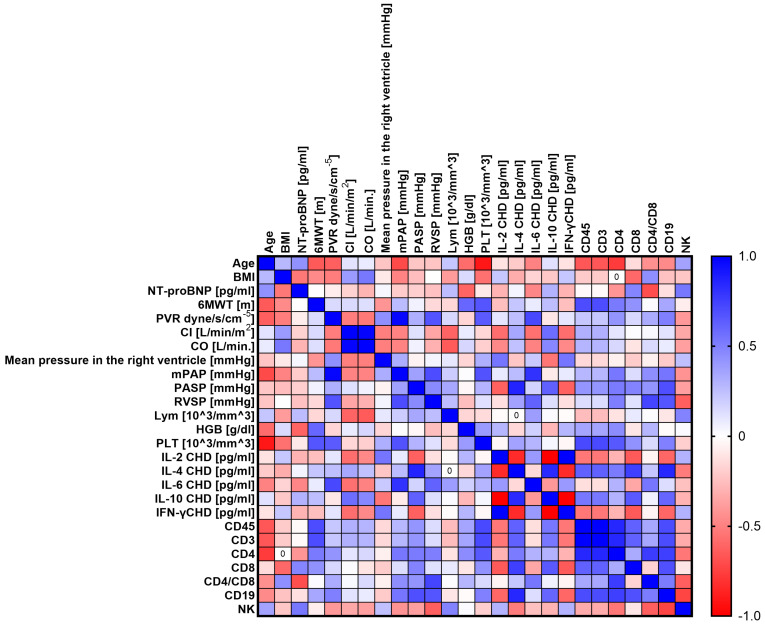
Graphical representation of Sperman’s rank correlation analysis for selected parameters of the immune system and clinical parameters in patients with CTD. Positive correlations are marked in blue and negative correlations in red. The intensity of the color is synonymous with the strength of the given correlation.

**Figure 7:**
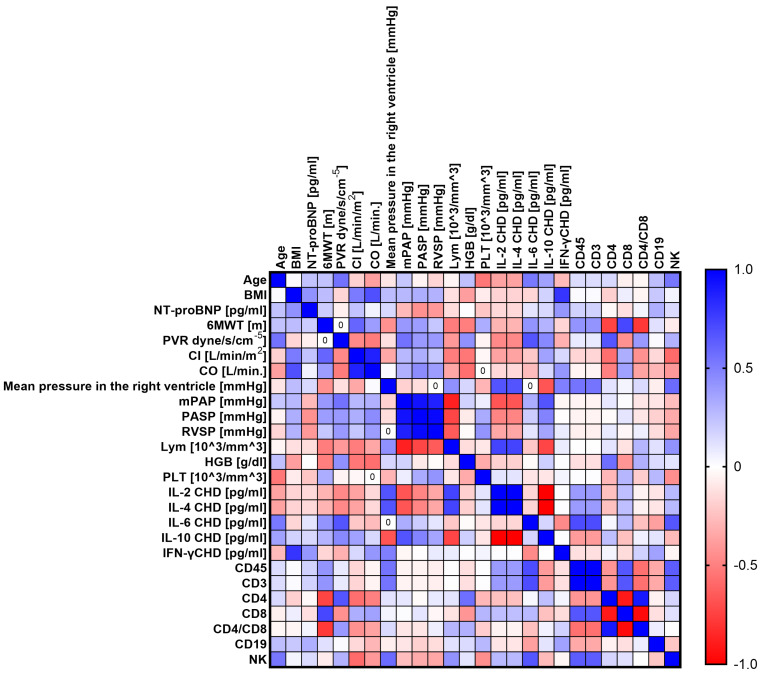
Graphical representation of Sperman’s rank correlation analysis for selected parameters of the immune system and clinical parameters in patients with CTEPH. Positive correlations are marked in blue and negative correlations in red. The intensity of the color is synonymous with the strength of the given correlation.

**Figure 8:**
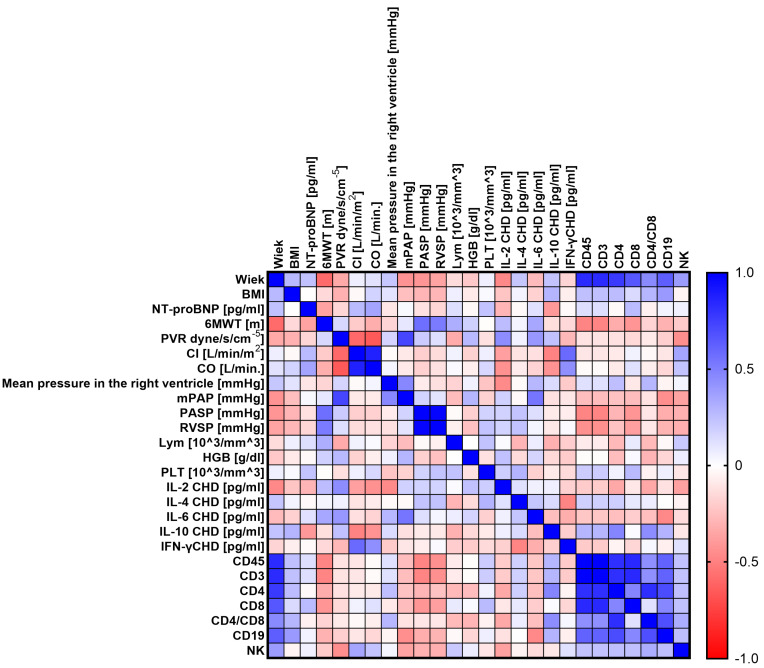
Graphical representation of Sperman’s rank correlation analysis for selected parameters of the immune system and clinical parameters in patients with IPAH. Positive correlations are marked in blue and negative correlations in red. The intensity of the color is synonymous with the strength of the given correlation.

The next step was to conduct analyses to show whether there are statistically significant correlations between the tested parameters of the immune system and the levels of the tested cytokines and selected clinical parameters of patients with PH. To this end, we performed a Sperman rank correlation analysis, which is graphically presented in Figure 5 (CHD), Figure 6 (CTD), Figure 7 (CTEPH), and Figure 8 (IPAH), and detailed research data are presented in Supplementary Materials Tables S2–S5.

Patients with CHD showed 46 statistically significant correlations, of which 13 were negative (1 low; 9 moderate; 3 high) and 33 positive (16 moderate, 13 high, and 4 very high). Patients with CTD showed 23 significant correlations, of which 5 were negative (2 high and 3 very high) and the remaining 18 were positive correlations (10 high and 8 very high). Then, for CTEPH patients, we recorded 28 correlations, of which 8 were negative (5 high and 3 very high) and 20 positive (15 high and 5 very high). In the last analyzed group of patients with IPAH, we noted 39 significant correlations, of which 13 were negative (12 moderate; 1 high) and 26 positive (14 moderate; 5 high; and 7 very high). A detailed analysis of statistically significant correlations between individual groups of patients showed several significant similarities between individual types of PH. For patients with CHD and CTD, we observed eight common correlations, namely age and mPAP (negative); PVR and RVSP (positive); mPAP and RVSP (positive); CD4+ and CD4+/CD8+ (positive); CD45+ and CD4+ (positive); CD4+ and CD3+ (positive); PVR and mPAP (positive); and age and PLT, which was positive in CHD patients but negative in CTD patients. 

For patients with CHD in relation to CTEPH, we also noted eight common correlations, namely CD45+ and CD8+ (positive); CD8+ and CD3+ (positive); mPAP and RVSP (positive); CD4+ and CD4+/CD8+ (positive); mPAP and PASP (positive); PASP and RVSP (positive); mPAP and lymphocytes, which was positive for CHD and negative for CTEPH; and PASP and lymphocytes, for which we recorded identical differences to those noted above. 

Between patients with CHD and IPAH, we distinguished the most common correlations, as many as 15, such as age and 6MWT (negative); age and mPAP (negative); age and PASP (negative); 6MWT and RVSP (positive); 6MWT and PASP (positive); CD19+ and CD4+/CD8+ (positive); PVR and mPAP (positive); CD45+ and CD4+ (positive); CD4+ and CD4+/CD8+ (positive); CD45+ and CD8+ (positive); ASP and RVSP (positive); and CO and IL-2 concentration and NT-proBNP and IL-10 concentration, for both of which the correlations were positive in patients with CHD and negative in patients with IPAH. Between patients with CTD and CTEPH, we found only two common correlations, both positive: Cl and CO and CD4+ and CD4+/CD8+.

In patients with CTD and IPAH, we detected 11 common correlations, the first 2 of which were negative and the rest positive. This applies to IL-4 and IFN-γ concentration; age and mPAP; PVR and IL-6 concentration; CD8+ and CD19+; mPAP and IL-6 concentration; CD45+ and CD19+; CD19+ and CD3+; CD4+ and CD19+; PVR and mPAP; CD45+ and CD4+; and CD3+ and CD4+.

Between the last groups of patients, i.e., CTEP and IPAH, we recorded six common coalescences, namely mean pressure in the right ventricle and IL-2 concentration (negative); CD4+ and CD4+/CD8+ (positive); CD3+ and CD8+ (positive); CO and Cl (positive); PASP and RVSP (positive); and CD4+ and CD8+, which was positive in IPAH patients and negative in CTEPH patients.

## 3. Discussion

### 3.1. IL-6

Many authors have considered the excessive production of cytokines as a potential cause for the development of pulmonary arterial hypertension (PAH) [9,10]. Studies have confirmed that patients with PAH of various causes have significantly higher levels of cytokines in their plasma compared with control groups, including IL-2, IL-6, IL-8, and IL-10, which have been shown to significantly impact patient survival and can serve as useful biomarkers for assessing the risk of developing PAH [11]. Additionally, a study conducted on patients with hereditary PAH revealed a higher risk of death in those with higher concentrations of IL-1α, IL-1β, IL-6, TNF-α, and IL-13, independent of other factors such as age, 6 min walk test results, cardiac output, and pressure in the right atrium [12]. These findings suggest that an increase in cytokine concentration is associated with patient mortality in PAH. IL-6 is a cytokine that plays a significant role in vascular remodeling and the development of PAH. Studies have shown that high levels of IL-6 are often associated with poor prognosis in patients with PAH. IL-6 is produced by various cells in response to stimulation and has both proinflammatory and anti-inflammatory effects. It can stimulate the production of acute phase proteins, promote B cell activation, and inhibit IFN-gamma production. However, IL-6 can also convert Th cells to Th2 and induce a suppressor of cytokine signaling-3 (SOCS-3), which negatively regulates the inflammatory response [11,13,14,15]. 

Research has found a correlation between IL-6 and the development of hypoxia-induced pulmonary hypertension, but some studies have not shown a direct correlation between IL-6 concentration and mPAP or PVR indices [16]. Our study assessed cytokine concentrations in patients with PAH caused by various factors or unknown causes. We found that the highest concentration of IL-6 was observed in patients with IPAH, which was significantly higher than in the control group. Although IL-6 concentration is associated with mortality risk in PAH patients, it is not necessarily an indicator of heart failure.

### 3.2. IL-2 

Research has shown that patients with chronic heart failure often have increased levels of IL-2 and IL-1. In animal studies, it was discovered that IL-2 can cause pulmonary edema by increasing vascular permeability. Additionally, IL-2 has been found to promote vasoconstriction and pulmonary hypertension. Studies have also confirmed that IL-2 is involved in the pathogenesis of IPAH, as it increases the expression of endothelin associated with the development of pulmonary hypertension [17,18,19]. Our research found that patients with IPAH had significantly higher concentrations of IL-2 compared with other PAH groups and the control group, as well as higher plasma IFN-gamma concentrations. Furthermore, patients with CHD-PAH, CTD-PAH, and CTEPH also showed significantly higher concentrations of IFN-gamma compared with the control group.

### 3.3. IL-10 

According to Soon et al.’s research, patients with IPAH have higher concentrations of IFN-gamma and IL-2, as well as IL-10 and IL-4, compared with the control group [13]. IL-10 is a cytokine that has protective effects on blood vessels and is produced by Th2 lymphocytes. It can inhibit the activation of Th1 lymphocytes and the production of proinflammatory cytokines by macrophages [20]. Furthermore, it prevents the proliferation of vascular cells, weakens smooth muscle cell proliferation, and reduces the expression of chemokines. IL-10 has also been found to lower the concentration of IL-6 [21]. Our studies have shown that patients with CHD-PAH, CTD-PAH, and CTEPH had significantly higher concentrations of IL-10 than the control group. However, there were no changes in the concentration of this cytokine in IPAH patients compared with the control group. Additionally, patients with CTD-PAH, CTEPH, and IPAH had significantly lower concentrations of IL-4 than the control group. Among the different types of PAH, IPAH patients had the lowest concentration of this cytokine. These results suggest that the anti-inflammatory component may not be functioning well in IPAH patients. The increased concentration of Th1-dependent cytokines (IFN-gamma, IL-2) with an increase in the concentration of IL-10 may be a compensatory effect that antagonizes inflammation [13]. Our research also indicated a disturbance in the function of Th2 lymphocytes in IPAH patients, where no increase in the concentration of anti-inflammatory cytokines was observed despite excessive secretion of proinflammatory cytokines.

### 3.4. IL-4

IL-4 is a cytokine that is linked with the development and progression of PAH. It has the ability to promote the production of antibodies by B lymphocytes, which is crucial in the development and progression of PAH. Studies have shown that patients with PAH have significantly higher levels of IL-4 in their plasma. However, in a separate research work, it was discovered that patients with CTD-PAH, CTEPH, and IPAH had significantly lower levels of IL-4 in their plasma compared with the controls. This suggests that the anti-inflammatory component may not be functioning properly. In addition, patients with CHD-PAH had higher levels of IL-4 compared with those with CTD-PAH, CTEPH, and IPAH. This is significant as IL-4 stimulates B lymphocytes to produce antibodies, which can contribute to the progression and development of PAH [22]. It is interesting to note that there is a difference of opinion regarding the correlation between cytokine levels and the risk of death in patients with hPAH (hereditary pulmonary arterial hypertension) when considering hemodynamic parameters. One study by Cracowski et al. [22] suggests that patients with hPAH who have elevated levels of cytokines (IL-1α, IL-1β, IL-6, IL-13, and TNF-α) have a higher risk of death, with cytokines identified as one of the independent factors affecting patient mortality. However, another study by Soon et al. [11] suggests that although inflammatory cytokines (TNF-a, IL-1b, IL-2, IL-4, IL-5, IL-6, IL-8, IL-10, IL-12, IL-13, and interferon-c) are involved in the pathogenesis of IPAH, there is no relationship between cytokine levels and hemodynamic parameters in hPAH patients. The disagreement may stem from the use of different parameters to assess the increased risk of death. Cracowski et al. employed age, the results of a walk test, RAP (right atrium pressure), and CO (cardiac output) [23], while Soon et al. used mPAP, CI (cardiac index), and PVR (pulmonary vascular resistance) as hemodynamic parameters [13]. According to a study by Yamaji-Kegan and colleagues, there is a theory that the inflammatory response to hypoxia-induced mitogenic factor (HIMF) is dependent on IL-4 and can cause inflammation in the lungs. The study found that administering HIMF protein resulted in VEGF-mediated lung inflammation through an IL-4-dependent pathway. HIMF increases VEGF expression and macrophage influx while decreasing VEGF receptor 2 expression. Previous research showed that mice without IL-4 did not experience this process, supporting the theory. Additionally, when the immune system responds to HIMF administration, it increases IL-4 production, which can cause pulmonary vascular pathway apoptosis [24,25,26]. The interaction between the protein cluster of differentiation 200, known as CD200, and the CD200R receptor is crucial in regulating the body’s inflammatory response [27]. This pathway helps to control inflammation during infections or cancer and can also suppress the immune system’s response to autoimmune diseases [28]. When CD200 interacts with the CD200R receptor, it can increase the production of cytokines in Th2 lymphocytes and alter the balance between Th1 and Th2 lymphocytes [29]. Lymphocytes stimulated with PHA and IL-2 show a higher incidence of CD200 on CD4+ T cells than on CD8+ T cells [30]. CD4+ T cells are responsible for suppressing excessive immune responses, which helps maintain immune system homeostasis. This CD200-CD200R system’s ability to reduce the immune system’s excessive response has been documented in many inflammatory diseases [31]. Furthermore, research has shown a negative correlation between the expression of the CD200R molecule on macrophages and CRP levels in rheumatoid arthritis [32]. In the authors’ research study, it was discovered that patients with CHD-PAH, CTEPH, and IPAH had higher percentages of certain T cells (CD4+ and CD8+) compared with the control group. This increase in T cells was found to be a compensatory mechanism that reduces excessive cytokine production. However, the study also revealed that this immunosuppressive pathway is impaired in patients with PAH. Additionally, the percentage of lymphocytes with certain expression was significantly lower in each PAH group compared with the control group, and IL-4 levels were lower in all PAH patients [4]. According to a study conducted by van Uden and colleagues, patients with IPAH have a reduced ability to produce certain substances, including IL-4, compared with healthy individuals [33]. This deficiency in the CD200R antigen, along with decreased IL-4 levels, suggests a potential link between the two conditions [27,34]. Other studies have also supported this hypothesis and have shown that IL-4 can increase the expression of CD200 and CD200R, as seen in microglia. In addition, research on Alzheimer’s disease and epilepsy in children has also found a correlation between IL-4 and CD200/CD200R expression [35].

### 3.5. IFN-Gamma

Cells produce interferons in response to certain triggers, and there are three types of interferons identified so far: type I (α, β, ε, κ, ω), type II (γ), and type III (λ) [36]. IFN is mainly produced by T lymphocytes, NK cells, NKT cells, and activated macrophages when they are influenced by antigens, cytokines, or mitogens. Although interferons do not have direct antiviral effects, they help induce an antiviral state in the cell. To function, interferons need to attach to the membrane receptor for IFN. The main antiviral function of interferons is to stimulate the synthesis of oligoisoadenine synthetase, which promotes the formation of adenine nucleotides in the presence of double-stranded RNA. These nucleotides activate the endoribonuclease RNase L, which degrades RNA, including cellular RNA [36]. When cells are treated with interferon at low concentrations, they produce more interferon than cells that are not treated with interferon. This is called interferon priming. According to Hemmerich et al. [37], interferon priming is important in causing neurotoxic phenotypes of specific tissue-resident macrophages (microglia). Interferon-γ also causes high release of IL-6, TNF-α, and nitric oxide, which can lead to a loss of electrical network activity and neurodegeneration. Even after interferon-γ disappears, its neurotoxic effects can still be active up to three days later. These findings are significant in addressing brain diseases, such as Alzheimer’s disease, viral and bacterial infections, and multiple sclerosis, which are characterized by elevated interferon-γ levels [37].

Dhillon, S. et al. [38] have presented four cases suggesting that pulmonary arterial hypertension (PAH) in patients who have been treated with interferon (IFN) may be irreversible. Therefore, clinicians should be cautious of this side effect when treating IFN, especially in patients with exertional dyspnea and no other identifiable cause of PAH [4]. Other studies have also reported a correlation between IFN type I treatment and the development of PAH [39,40]. The European Society of Cardiology guidelines for the diagnosis and treatment of pulmonary hypertension [41] further support this relationship by listing interferon treatment, along with cocaine, phenylpropanolamine, St John’s wort, amphetamine-like drugs, and certain chemotherapeutic agents, as possible causes of PAH. Research also suggests that the diffusing capacity of the lung for carbon monoxide (DLCO) frequently declines in patients with HCV who are treated with interferons and ribavirin. Specifically, clinically significant reductions in DLCO were observed in 48% of all patients treated with interferon (Peg-IFNa-2a) and ribavirin for HCV, and 18% of these patients still had a significant reduction in DLCO up to 6 months post-therapy, which aligns with earlier reports [42]. Research suggests that IFN-γ, which has antiviral and antitumor properties, may also play a significant role in PH [13]. Studies by Soon et al. with a separate cohort of 25 patients with IPAH found noticeably higher levels of IFN-γ in IPAH patients compared with control groups [43]. Proinflammatory cytokines like IL-2 and IL-6 were also found to be significantly elevated in IPAH patients. The median IFN-γ value in the IPAH group was almost six times higher than in the healthy control group, with the highest concentration levels observed in IPAH patients. Additionally, patients with CHD-PAH, CTD-PAH, and CTEPH were found to have higher IFN-γ concentrations compared with the control group in another study [4]. 

According to van Uden et al., T-helper cells in patients with IPAH were less capable of producing certain cytokines compared with those in healthy individuals. Specifically, IPAH patients had significantly decreased levels of TNFα, IFN-γ, IL-4, and IL-17 in CD4+ and CD8+ T cells. Additionally, IPAH patients had reduced proportions of certain double-producing T cells and lower frequencies of certain single-producing and double-producing memory T cells. However, CTD-PAH patients did not show these differences in cytokine production, even those who had received immunomodulatory therapy. The study also found that IPAH patients had reduced frequencies of Th2 cells, but the proportions of other cytokine-producing T cells remained the same as in healthy individuals. The researchers noted that the reduced cytokine production was only observed in memory T cells of IPAH patients and not in CTD-PAH patients [33]. It is possible that patients with a lower percentage of cytokine-producing cells may have a greater likelihood of developing IPAH. This conclusion is based on observations of patients with COPD, where those in the early stages of the disease had higher proportions of IFN-γ + and TNF-α + CD8+ T cells, while those in advanced stages had lower levels of IL-17+ CD4+ T cells. In CETPH patients, high levels of CXCL9 chemokine and IL-8 at the start were linked to increased mortality. CXCL9 is a chemokine that regulates immune cell activity, particularly T1 cells, and is necessary for IFN-γ production. T17.1 cells, which produce IFN-γ, are identified by CXCR3, a receptor for CXCL9. T17 cell immune polarization was discovered in patients with PAH, while intravascular triggers in CTEPH patients may stimulate CXCL9 to recruit cytotoxic lymphocytes, natural killer cells, and macrophages. CXCL9 may therefore be a significant biomarker that reflects the immune system’s pathological involvement in the pathophysiology of CTEPH [5,44,45].

## 4. Materials and Methods

### 4.1. Examined Patients and the Control Group Characteristics

**Figure 9:**
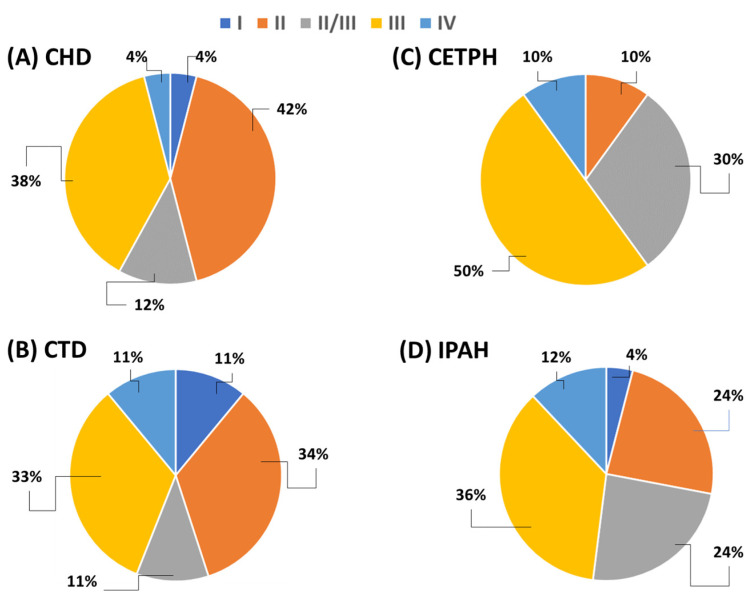
Percentage of patients in a defined class of heart failure (according to WHO), in particular with types of pulmonary arterial hypertension.

This research was carried out between October 2017 and February 2018 on 70 patients with pulmonary arterial hypertension (50 women and 20 men), patients of the Cardiology Department of the Medical University of Lublin and the Cardiology Department of the Provincial Specialist Hospital in Lublin. The diagnosis of pulmonary arterial hypertension was based on the criteria of the European Society of Cardiology [41]. The average age of the patients was 57.74 ± 17.17 years (median: 60 years, minimum: 23 years, maximum: 81 years). Patients were classified according to the type of pulmonary hypertension, as follows: chronic thromboembolic pulmonary hypertension (CTEPH); pulmonary arterial hypertension associated with congenital heart disease (CHD); pulmonary arterial hypertension connected with systemic tissue diseases (CTDs); and idiopathic pulmonary arterial hypertension (IPAH). Detailed data on the characteristics of individual patient groups are presented in Table 4. The percentage of patients in an established class of heart failure (according to the World Health Organization), in particular with types of pulmonary arterial hypertension, is presented in Figure 9.

This study was conducted on people who did not present clinical symptoms of infection, allergies, or autoimmune diseases and who had not undergone a blood transfusion in the two months preceding the study. Basic laboratory tests were executed in the ALAB laboratory of the Independent Public Clinical Hospital in Lublin. Transthoracic heart echocardiography (TTE) was also performed using the Philips iE33 devices owned by the Cardiology Department of the Medical University of Lublin, the Cardiac Catheterization Laboratory of the Independent Public Clinical Hospital, and the Department of Cardiology of the Provincial Specialist Hospital in Lublin according to the standards of hemodynamic and angiographic assessment of pulmonary circulation adopted by the Circulatory Section and the Association of Cardiovascular Interventions of the Polish Society of Cardiology [23]. Due to the invasive nature of cardiac catheterization, the last available test result was used for analysis. The hemodynamic examination was performed on the analyzed patients depending on their clinical condition (based on the ESC guidelines on monitoring patients with PAH) and based on the requirements of the national PAH therapeutic program (at least every 24 months, and in patients with Eisenmenger syndrome, once in a lifetime).

The control group consisted of 20 people (12 women and 8 men) aged 58.1 ± 11.1 years (median: 56 years, minimum: 39 years, maximum: 77 years). Among the volunteers, only people who had no history of cardiovascular disease, were not treated with preparations affecting the immune system, did not show signs of infection, autoimmune diseases, or allergies, and did not have blood transfusions were selected.

The study protocol received a positive opinion from the Bioethics Committee at the Medical University of Lublin (no. KE-0254/309/2016).

### 4.2. Tested Material: Assessment of Peripheral Blood Immunophenotype and Plasma Interleukin Concentration

The material for the study was peripheral blood, which was collected from patients with pulmonary arterial hypertension and the control group. In the course of the work, 10 mL of blood was collected in tubes containing EDTA in a vacuum-aspiration system (Sarstedt, Germany). The collected blood was used to assess the immunophenotype of all tested patients and to separate the plasma for further analysis. 

**Figure 10:**
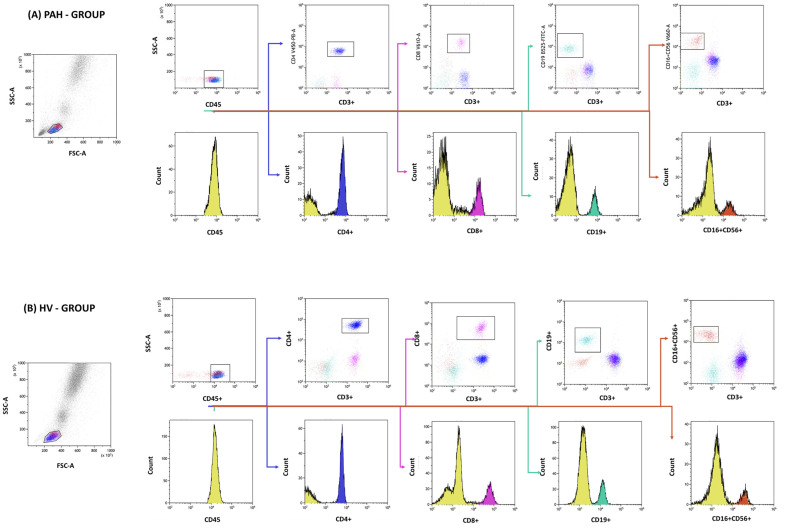
An example of the gating strategy adopted for the study patients (**A**) and healthy volunteers (**B**). The gating strategy involves evaluating subsets of T and B lymphocytes and natural killer cells using dot plots and histograms. In particular, events manifesting the phenotypes CD4+ (blue), CD8+ (purple), CD19+ (sea color), and CD16+CD56+ (brown) were quantified.

We used flow cytometry to assess the immunophenotype of lymphocytes in peripheral blood. Specifically, a whole blood sample was collected and stained with a panel of monoclonal antibodies, which included anti-CD4 BV421 (562424, BD Bioscience, Franklin Lakes, NJ, USA), anti-CD3 PerCp (345766, BD Bioscience, USA), anti-CD8 BV605 (344742, BioLegend, San Diego, CA, USA), anti-CD19 FITC (555412, BD Bioscience, USA), anti-CD45 Alexa Fluor 700 (368514, BioLegend, USA), anti-CD56 BV650 (564057, BD Bioscience, USA), and anti-CD16 BV650 (555407, BD Bioscience, USA). After staining, a lysing buffer was applied to remove red blood cells, and the resulting cells were washed and analyzed using a Cytoflex LX instrument (Beckman Coulter, Brea, CA, USA). Data analysis was performed using the Kaluza Analysis program (Beckman Coulter, USA) (Figure 10).

This process allowed for a detailed examination of the lymphocyte population in the blood sample, revealing important insights into its overall immunophenotype. The concentrations of IFN-gamma, IL-2, IL-4, IL-6, and IL-10 were determined in the peripheral blood plasma of patients from the study and control groups with ELISA (enzyme-linked immunosorbent assay). The following sets were used for the determinations:

- Human IFN-gamma Platinum ELISA, sensitivity = 0.99 pg/mL (eBioscience, San Diego, CA, USA);
- Human IL-2 Quantikine ELISA Kit, sensitivity = 0.066 pg/mL (R&D Systems, Minneapolis, MN, USA);
- Human IL-4 Quantikine ELISA Kit, sensitivity = 0.22 pg/mL (R&D Systems, USA);
- Human IL-10 Quantikine ELISA Kit, sensitivity = 0.17 pg/mL (R&D Systems, USA);
- Human IL-6 Quantikine ELISA Kit, sensitivity = 0.11 pg/mL (R&D Systems, USA).

Cytokine concentration was determined according to the manufacturer’s procedure. The light absorbance was read using a VICTOR automatic reader (Perkin Elmer, Waltham, MA, USA). Calculation of the concentrations of the tested samples was performed using the WorkOut computer program based on the drawn standard curve.

### 4.3. Statistical Analysis

Data analysis was conducted using Tibco Statistica 13.3 software from StatSoft. The Shapiro–Wilk test determined the normal distribution of continuous variables. Obtained values were expressed as mean and standard deviation (SD) and median and minimum and maximum. Independent variables were compared using both the *t*-test and the Mann–Whitney U test to assess intergroup differences. Differences among more groups were assessed using the Kruskal–Wallis test, followed by post hoc analysis of mean ranks for multiple comparisons. Correlation analyses were conducted using Sperman rank correlation analysis. Statistically significant results were indicated by *p* ≤ 0.05. 

## 5. Conclusions

This study by Tomaszewski M et al. found that patients with CHD-PAH, CTD-PAH, and CTEPH had higher IL-6, IFN-gamma, and IL-2 levels than the control group. However, they also had elevated IL-10 concentrations, which could indicate an effort to counteract inflammation. Patients with IPAH had even higher levels of IL-6, IFN-gamma, and IL-2 than other PAH types, but lower levels of IL-10. Other researchers’ findings were either consistent or conflicting with these results. It is widely recognized that cytokines play a crucial role in PH development. However, further research is required to determine whether cytokines can be used as diagnostic markers or potential treatment targets for this disease.

## Figures and Tables

**Table 1:** Analysis of the peripheral blood immunophenotypes of patients with PAH in relation to healthy volunteers.

Parameters	Study Group (*n* = 70)		Healthy Volunteers (*n* = 20)		*p*-Value
	Mean ± SD	Median (Range)	Mean ± SD	Median (Range)	
CD45+ [%]	75.19 ± 3.20	74.58 (62.81–81.57)	96.27 ± 0.73	96.23 (95.20–97.87)	0.000 *
CD3+ [%]	60.91 ± 2.59	60.41 (45.20–68.93)	81.39 ± 0.98	81.42 (80.00–82.74)	0.000 *
CD4+ [%]	35.54 ± 1.95	35.73 (25.64–41.23)	48.73 ± 3.38	48.38 (44.11–54.13)	0.000 *
CD8+ [%]	25.37 ± 2.98	25.36 (16.53–34.24)	32.66 ± 3.41	33.13 (27.75–38.48)	0.000 *
Ratio of CD4+/CD8+	1.43 ± 0.23	1.37 (0.83–2.04)	1.52 ± 0.26	1.44 (1.15–1.95)	0.429
CD19+ [%]	5.93 ± 0.58	5.83 (3.89–7.25)	7.94 ± 0.66	7.96 (7.04–8.93)	0.000 *
NK [%]	3.53 ± 0.84	3.40 (1.98–5.02)	7.13 ± 0.65	7.34 (6.19–7.98)	0.000 *

Statistically significant results are marked with *.

**Table 2:** Peripheral blood immunophenotype analysis of patients with CTEPH, CHD, CTD, and IPAH in relation to healthy volunteers.

Parameters	CTEPH (*n* = 10)		CHD (*n* = 26)		CTD (*n* = 9)		IPAH (*n* = 25)		Healthy Volunteers (*n* = 20)		*p*-Value	*p*-Value									
	Mean ± SD	Median (Range)	Mean ± SD	Median (Range)	Mean ± SD	Median (Range)	Mean ± SD	Median (Range)	Mean ± SD	Median (Range)		CHD vs. CTEPH	CHD vs. CTD	CHD vs. IPAH	CTEPH vs. CTD	CTEPH vs. IPAH	CTD vs. IPAH	CHD vs. HV	CTEPH vs. HV	CTD vs. HV	IPAH vs. HV
CD45+ [%]	65.77 ± 2.34	64.29 (62.81–71.93)	69.11 ± 3.93	69.08 (60.01–76.67)	65.21 ± 5.67	64.97 (55.80–75.74)	71.37 ± 8.03	74.26 (56.53–85.10)	96.27 ± 0.73	96.23 (95.20–97.87)	0.000 *	0.006 *	0.057	0.199	0.905	0.07	0.072	0.000 *	0.000 *	0.000 *	0.000 *
CD3+ [%]	53.27 ± 1.90	52.08 (50.87–58.26)	55.98 ± 3.16	55.95 (48.61–62.10)	52.82 ± 4.59	52.63 (45.20–61.35)	57.81 ± 6.51	60.15 (45.79–68.93)	81.39 ± 0.98	81.42 (80.00–82.74)	0.000 *	0.006 *	0.057	0.199	0.905	0.07	0.072	0.000 *	0.000 *	0.000 *	0.000*
CD4+ [%]	30.73 ± 1.34	30.65 (29.28–33.31)	32.67 ± 2.07	32.87 (28.68–37.14)	30.60 ± 2.89	30.84 (25.64–36.69)	33.73 ± 4.09	34.69 (26.38–41.23)	48.73 ± 3.38	48.38 (44.11–54.13)	0.000 *	0.011 *	0.016 *	0.281	0.905	0.064	0.072	0.000 *	0.000 *	0.000 *	0.000 *
CD8+ [%]	22.54 ± 2.76	21.43 (17.56–28.94)	23.34 ± 3.04	23.14 (17.96–30.85)	22.22 ± 3.40	21.79 (17.74–30.48)	24.11 ± 3.81	24.46 (16.53–34.24)	32.66 ± 3.41	33.13 (27.75–38.48)	0.000 *	0.392	0.171	0.437	0.604	0.211	0.151	0.000 *	0.000 *	0.000 *	0.000 *
Ratio of CD4+/CD8+	1.23 ± 0.19	1.30 (0.92–1.65)	1.31 ± 0.22	1.26 (0.90–1.84)	1.24 0.24	1.21 (0.91–1.82)	1.36 ± 0.28	1.35 (0.83–2.04)	1.52 ± 0.26	1.44 (1.15–1.95)	0.023 *	0.392	0.402	0.786	0.905	0.339	0.376	0.000 *	0.005 *	0.011 *	0.053
CD19+ [%]	5.09 ± 0.55	4.62 (4.36–6.12)	5.46 ± 0.55	5.34 (4.46–6.53)	5.15 ± 0.80	5.09 (3.87–6.45)	5.62 ± 0.84	5.54 (4.10–7.25)	7.94 ± 0.66	7.96 (7.04–8.93)	0.000 *	0.109	0.342	0.507	0.905	0.097	0.216	0.000 *	0.000 *	0.000 *	0.000 *
NK [%]	3.05 ± 0.74	2.61 (1.86–4.18)	3.22 ± 0.80	3.15 (1.90–4.53)	3.11 ± 0.50	3.12 (1.98–3.83)	3.35 ± 0.83	3.33 (1.75–5.02)	7.13 ± 0.65	7.34 (6.19–7.98)	0.000 *	0.475	0.753	0.647	0.780	0.460	0.618	0.000 *	0.000 *	0.000 *	0.000 *

Statistically significant results are marked with *.

**Table 3:** Evaluation of the concentration of tested cytokines in patients with PAH in relation to healthy volunteers.

Cytokine Concentration [pg/mL]	Study Group (*n* = 70)		Healthy Volunteers (*n* = 20)		*p*-Value
	Mean ± SD	Median (Range)	Mean ± SD	Median (Range)	
IL-2	18.82 ± 4.86	18.12 (4.30–50.12)	2.76 ± 1.00	2.36 (0.48–7.16)	0.000 *
IL-4	4.35 ± 1.56	4.25 (0.64–14.33)	4.80 ± 0.31	4.67 (4.29–5.31)	0.236
IL-6	30.52 ± 9.63	29.53 (9.35–60.30)	4.13 ± 2.11	3.17 (0.15–17.20)	0.000 *
IL-10	10.87 ± 2.91	10.62 (2.07–35.30)	4.06 ± 1.02	4.08 (2.77–6.16)	0.000 *
IFN-γ	5.95 ± 1.41	6.02 (0.40–23.30)	2.38 ± 1.19	2.34 (0.61–4.38)	0.000 *

Statistically significant results are marked with *.

**Table 4:** Detailed data on the characteristics of individual patient groups with PH.

Parameters	CHD (*n* = 26)		CTEPH (*n* = 10)		CTD (*n* = 9)		IPAH (*n* = 25)		*p*-Value	*p*-Value					
	Mean ± SD	Median (Range)	Mean ± SD	Median (Range)	Mean ± SD	Median (Range)	Mean ± SD	Median (Range)		CHD vs. CTEPH	CHD vs. CTD	CHD vs. IPAH	CTEPH vs. CTD	CTEPH vs. IPAH	CTD vs. IPAH
Sex	7 males 19 females		3 males 7 females		0 males 9 females		10 males 5 females								
Age	55.69 ± 17.00	57.00 (23.00–81.00)	71.10 ± 8.40	72.40 (54.00–81.00)	52.22 ± 17.62	54.00 (28.00–77.00)	56.52 ± 16.88	62.00 (23.00–81.00)	0.043 *	0.014 *	0.724	0.829	0.010 *	0.013 *	0.489
BMI	25.54 ± 4.10	24.90 (19.50–38.15)	24.74 ± 3.97	23.67 (20.44–35.04)	26.35 ± 6.93	25.70 (20.32–44.44)	27.53 ± 9.67	26.00 (17.10–40.52)	0.318	0.475	0.867	0.186	0.905	0.090	0.355
NT-proBNP [pg/mL]	1507.04 ± 1898.37	804.00 (106.80–9350.00)	1899.62 ± 1635.75	1462.50 (53.00–5991.00)	734.94 ± 436.55	454.00 (127.00–1469.00)	1192.47 ± 1019.51	1076.00 (73.98–3895.00)	0.391	0.271	0.402	0.933	0.095	0.286	0.316
6MWT [m]	323.48 ± 153.14	378.00 (0.00–578.00)	361.80 ± 122.44	301.00 (202.00–539.00)	358.15 ± 156.04	420.00 (80.00–577.50)	381.04 ± 100.29	374.00 (136.00–556.00)	0.734	0.505	0.564	0.316	0.905	0.578	0.939
PVR [dyne/s/cm^−5^]	1317.12 ± 812.60	1123.43 (134.00–2803.00)	723.89 ± 233.86	742.00 (305.51–1125.80)	424.53 ± 348.41	355.00 (139.00–1292.00)	697.60 ± 308.26	651.00 (158.00–1599.00)	0.001 *	0.072	0.001 *	0.005 *	0.04 *	0.645	0.017 *
CI [L/min/m^2^]	2.40 ± 1.07	2.10 (1.65–7.32)	2.59 ± 1.23	2.10 (1.75–5.80)	3.16 ± 0.78	3.21 (1.83–4.67)	2.54 ± 0.64	2.60 (1.43–3.75)	0.02 *	0.838	0.003 *	0.055	0.094	0.355	0.055
CO [L/min]	4.01 ± 2.08	3.52 (2.29–13.90)	4.37 ± 2.09	3.72 (2.48–9.51)	5.37 ± 1.48	5.76 (3.02–8.47)	4.64 ± 1.13	4.46 (2.11–6.42)	0.003 *	0.955	0.002 *	0.002 *	0.094	0.188	0.298
Mean pressure in the right ventricle [mmHg]	8.00 ± 3.27	8.00 (1.00–16.00)	9.11 ± 4.86	7.00 (3.00–18.00)	8.56 ± 3.80	8.00 (3.00–15.00)	8.80 ± 5.58	9.00 (2.00–23.00)	0.994	0.867	0.955	0.874	0.931	0.730	0.878
mPAP [mmHg]	59.15 ± 26.64	51.00 (26.00–106.00)	45.85 ± 8.74	46.00 (25.60–56.00)	35.30 ± 12.49	34.00 (25.00–68.00)	45.24 ± 12.43	48.00 (17.00–66.00)	0.038 *	0.491	0.005 *	0.026	0.019 *	0.908	0.06
PASP [mmHg]	88.04 ± 32.77	83.00 (41.00–150.00)	82.00 ± 20.97	85.00 (39.00–110.00)	62.56 ± 18.58	61.00 (35.00–110.00)	71.48 ± 20.53	77.00 (37.00–96.00)	0.085	0.697	0.034 *	0.092	0.024 *	0.270	0.442
RVSP [mmHg]	91.88 ± 33.16	100.00 (47.00–150.00)	81.56 ± 17.59	80.00 (50.00–110.00)	64.22 ± 19.94	61.00 (36.00–115.00)	72.44 ± 20.29	79.00 (42.00–96.00)	0.053	0.445	0.046 *	0.029 *	0.040 *	0.298	0.514
Lymphocytes [10^3^/mm^3^]	1.72 ± 0.46	1.62 (1.10–2.77)	2.51 ± 0.91	2.43 (1.30–3.83)	2.47 ± 0.38	2.60 (1.67–3.04)	2.15 ± 0.55	2.01 (1.20–3.14)	0.002 *	0.03 *	0.000 *	0.006 *	0.905	0.321	0.120
Hemoglobin [g/dL]	15.40 ± 4.30	15.70 (7.40–22.10)	13.24 ± 2.51	13.75 (8.50–16.70)	13.76 ± 2.28	13.50 (11.30–19.40)	13.66 ± 2.03	13.60 (9.50–18.50)	0.491	0.200	0.424	0.205	0.968	0.928	0.818
Platelet count [10^3^/mm^3^]	164.11 ± 63.13	153.00 (62.00–299.00)	189.00 ± 69.38	182.50 (93.00–348.00)	147.88 ± 87.08	114.00 (55.00–309.00)	211.40 ± 78.95	210.00 (79.00–474.00)	0.048 *	0.355	0.271	0.022 *	0.156	0.377	0.037 *

NT-proBNP—N-terminal pro–B-type natriuretic peptide; 6MWT—6 min walk test; PVR—pulmonary vascular resistance; CI—cardiac index; CO—cardiac output; mPAP—mean pulmonary artery pressure; PASP—pulmonary arterial systolic pressure; RVSP—right ventricular systolic pressure. Statistically significant results are marked with *.

## Data Availability

Due to privacy and ethical concerns, the data that support the findings of this study are available on request from the first author (M.T.).

## Supplementary Materials

The supporting information can be downloaded at https://www.mdpi.com/article/10.3390/ijms241813694/s1.
