# Supplementary material for: In the Search for Biomarkers of Pulmonary Arterial Hypertension, Are Cytokines IL-2, IL-4, IL-6, IL-10, and IFN-Gamma the Right Indicators to Use?
Source: Int J Mol Sci. 2023 Sep 5;24(18):13694. doi: 10.3390/ijms241813694 (PMC10530884; doi:10.3390/ijms241813694)
Supplement: Supplementary file 1 [file ijms-24-13694-s001.zip › ijms-2513775-supplementary.pdf]

Supplementary materials Table S1. Analysis of cytokine concentration in patients with CTEPH, CHD, CTD, IPAH in relation to healthy individuals.

**Type of PAH**		**Cytokine concentration [pg/ml]**				
		**IL-2**	**IL-4**	**IL-6**	**IL-10**	**IFN-γ**
**CTEPH**	Mean±SD	8.14± 0.44	2.87± 0.19	23.86± 6.47	6.93± 0.71	5.34± 0.18
	Median (range)	7.89 (7.74-8.82)	2.86 (2.61-3.21)	22.16 (11.12-31.23)	7.07 (5.93-7.99)	5.35 (5.06-5.57)
**CHD**	Mean±SD	29.16± 9.67	6.09± 2.81	18.96± 8.51	19.84± 5.61	4.37± 2.06
	Median (range)	26.66 (16.30-50.12)	5.89 (1.67-12.24)	17.90 (9.35-30.46)	14.65 (3.95-35.30)	4.26 (0.40-10.03)
**CTD**	Mean±SD	5.82± 1.03	5.89± 2.56	33.10± 13.08	11.00± 1.56	4.88± 0.13
	Median (range)	6.09 (4.30-6.91)	4.69 (3.23-14.33)	30.97 (10.57-40.88)	10.68 (8.76-13.66)	4.93 (4.63-5.05)
**IPAH**	Mean±SD	32.19± 8.32	2.57± 0.67	46.66± 10.47	5.72± 3.78	9.20± 3.26
	Median (range)	23.80 (9.39-50.12)	1.74 (0.64-9.24)	45.96 (12.30-60.30)	4.91 (2.07-15.71)	8.04 (5.58-23.30)
**Healthy volunteers**	Mean±SD	2.76± 1.00	4.80± 0.31	4.13± 2.11	4.06± 1.02	2.38± 1.19
	Median (range)	2.36 (0.48-7.16)	4.67 (4.29-5.31)	3.17 (0.15-17.20)	4.08 (2.77-6.16)	2.34 (0.61-4.38)
p-value		0.000*	0.000*	0.000*	0.000*	0.000*
p-value	HV vs. CTEPH	0.000*	0.000*	0.000*	0.000*	0.072*
	CTEPH vs. CHD	0.000*	0.000*	0.031*	0.000*	0.124
	CTEPH vs. CTD	0.032*	0.000*	0.028*	0.017*	0.277
	CTEPH vs. IPAH	0.000*	0.000*	0.000*	0.000*	0.000*
	HV vs. CHD	0.000*	0.027*	0.000*	0.000*	0.000*
	CHD vs. CTD	0.000*	0.048*	0.000*	0.011*	0.312
	CHD vs. IPAH	0.043*	0.000*	0.000*	0.000*	0.000*
	HV vs. CTD	0.000*	0.973	0.000*	0.000*	0.000*
	CTD vs. IPAH	0.000*	0.000*	0.013*	0.000*	0.000*
	HV vs. IPAH	0.000*	0.000*	0.000*	0.142	0.000*

Statistically significant results are marked with *.

Supplementary materials Table S2. Sperman rank correlation analysis for patients with CHD

**Parameters**	**R**	**t(N-2)**	**p-value**
**Wiek & Lym [10^3/mm^3]**	-0 .647	-4 .156	0 .000
**Wiek & RVSP [mmHg]**	-0 .644	-4 .124	0 .000
**Wiek & HGB [g/dl]**	-0 .604	-3 .710	0 .001
**Wiek & PASP [mmHg]**	-0 .572	-3 .415	0 .002
**Wiek & mPAP [mmHg]**	-0 .565	-3 .353	0 .003
**HGB [g/dl] & PLT [10^3/mm^3]**	-0 .463	-2 .561	0 .017
**Wiek & PVR dyne/s/cm-5]**	-0 .456	-2 .509	0 .019
**6MWT [m] & CO [L/min.]**	-0 .428	-2 .269	0 .033
**NT-proBNP [pg/ml] & HGB [g/dl]**	-0 .419	-2 .263	0 .033
**BMI & 6MWT [m]**	-0 .413	-2 .178	0 .040
**IL-2 [pg/ml] & NK**	-0 .413	-2 .224	0 .036
**CI [L/min/m2] & Mean pressure in the right ventricle**	-0 .412	-2 .217	0 .036

**[mmHg]**			
**Wiek & 6MWT [m]**	-0 .397	-2 .072	0 .050
**PVR dyne/s/cm-5] & HGB [g/dl]**	0 .400	2 .140	0 .043
**6MWT [m] & mPAP [mmHg]**	0 .414	2 .179	0 .040
**CD45 & CD4**	0 .454	2 .499	0 .020
**CD4 & CD3**	0 .454	2 .499	0 .020
**6MWT [m] & HGB [g/dl]**	0 .457	2 .464	0 .022
**PLT [10^3/mm^3] & IFN-? [pg/ml]**	0 .462	2 .551	0 .018
**PVR dyne/s/cm-5] & Lym [10^3/mm^3]**	0 .469	2 .600	0 .016
**6MWT [m] & PASP [mmHg]**	0 .469	2 .546	0 .018
**CO [L/min.] & IL-10 [pg/ml]**	0 .471	2 .615	0 .015
**PLT [10^3/mm^3] & Wiek**	0 .492	2 .768	0 .011
**PVR dyne/s/cm-5] & 6MWT [m]**	0 .497	2 .745	0 .012
**CD19 & CD4/CD8**	0 .516	2 .954	0 .007
**HGB [g/dl] & IL-2 [pg/ml]**	0 .518	2 .964	0 .007
**6MWT [m] & RVSP [mmHg]**	0 .533	3 .025	0 .006
**NT-proBNP [pg/ml] & IL-4 [pg/ml]**	0 .535	3 .103	0 .005
**NT-proBNP [pg/ml] & IL-10 [pg/ml]**	0 .540	3 .142	0 .004
**PASP [mmHg] & Lym [10^3/mm^3]**	0 .612	3 .789	0 .001
**PVR dyne/s/cm-5] & RVSP [mmHg]**	0 .642	4 .107	0 .000
**RVSP [mmHg] & Lym [10^3/mm^3]**	0 .643	4 .112	0 .000
**Lym [10^3/mm^3] & HGB [g/dl]**	0 .654	4 .230	0 .000
**PVR dyne/s/cm-5] & PASP [mmHg]**	0 .660	4 .307	0 .000
**PASP [mmHg] & HGB [g/dl]**	0 .670	4 .425	0 .000
**mPAP [mmHg] & HGB [g/dl]**	0 .672	4 .448	0 .000
**RVSP [mmHg] & HGB [g/dl]**	0 .719	5 .068	0 .000
**CD4 & CD4/CD8**	0 .740	5 .393	0 .000
**mPAP [mmHg] & RVSP [mmHg]**	0 .744	5 .454	0 .000
**mPAP [mmHg] & PASP [mmHg]**	0 .763	5 .780	0 .000
**mPAP [mmHg] & Lym [10^3/mm^3]**	0 .790	6 .318	0 .000
**PVR dyne/s/cm-5] & mPAP [mmHg]**	0 .793	6 .369	0 .000
**CD45 & CD8**	0 .826	7 .188	0 .000
**CD8 & CD3**	0 .826	7 .188	0 .000
**CI [L/min/m2] & CO [L/min.]**	0 .890	9 .586	0 .000
**PASP [mmHg] & RVSP [mmHg]**	0 .961	16 .962	0 .000

Supplementary materials Table S3 Sperman rank correlation analysis for patients with CTD

Parameters	R	t(N-2)	p-value
**Wiek & PLT [10^3/mm^3]**	-0 .916	-6 .040	0 .001
**IL-2 [pg/ml] & IL-4 [pg/ml]**	-0 .833	-3 .989	0 .005
**IL-4 [pg/ml] & IFN-γ [pg/ml]**	-0 .833	-3 .989	0 .005
**IL-2 [pg/ml] & IL-4 [pg/ml]**	-0 .833	-3 .989	0 .005
**IL-4 [pg/ml] & IFN-γ [pg/ml]**	-0 .833	-3 .989	0 .005
**CD19 & NK**	-0 .733	-2 .854	0 .025
**Wiek & mPAP [mmHg]**	-0 .713	-2 .687	0 .031

PVR dyne/s/cm-5] & RVSP [mmHg]	0 .672	2 .404	0 .047
mPAP [mmHg] & PLT [10^3/mm^3]	0 .678	2 .442	0 .045
CD8 & CD19	0 .683	2 .476	0 .042
mPAP [mmHg] & RVSP [mmHg]	0 .694	2 .553	0 .038
CD45 & CD19	0 .700	2 .593	0 .036
CD19 & CD3	0 .700	2 .593	0 .036
PVR dyne/s/cm-5] & IL-6 [pg/ml]	0 .717	2 .719	0 .030
IL-4 [pg/ml] & CD8	0 .750	3 .000	0 .020
CD4 & CD4/CD8	0 .750	3 .000	0 .020
CD4 & CD19	0 .750	3 .000	0 .020
mPAP [mmHg] & IL-6 [pg/ml]	0 .804	3 .573	0 .009
IL-4 [pg/ml] & IL-10 [pg/ml]	0 .833	3 .989	0 .005
IL-4 [pg/ml] & CD19	0 .850	4 .269	0 .004
CD45 & CD4	0 .850	4 .269	0 .004
CD4 & CD3	0 .850	4 .269	0 .004
PASP [mmHg] & IL-4 [pg/ml]	0 .863	4 .511	0 .003
PVR dyne/s/cm-5] & mPAP [mmHg]	0 .975	11 .533	0 .000
CI [L/min/m2] & CO [L/min.]	0 .983	14 .248	0 .000

Supplementary materials Table S4 Sperman rank correlation analysis for patients with CTEPH

Parameters	R	t(N-2)	p-value
CD8 & CD4/CD8	-0 .964	-10 .200	0 .000
CD4 & CD8	-0 .903	-5 .946	0 .000
mPAP [mmHg] & Lym [10^3/mm^3]	-0 .874	-4 .758	0 .002
IL-10 [pg/ml] & Lym [10^3/mm^3]	-0 .729	-3 .017	0 .017
Lym [10^3/mm^3] & PASP [mmHg]	-0 .728	-2 .810	0 .026
IL-2 [pg/ml] & mPAP [mmHg]	-0 .669	-2 .384	0 .049
IL-4 [pg/ml] & mPAP [mmHg]	-0 .669	-2 .384	0 .049
IL-10 [pg/ml] & Mean pressure in the right ventricle [mmHg]	-0 .669	-2 .384	0 .049
CD45 & NK	0 .636	2 .333	0 .048
NK & CD3	0 .636	2 .333	0 .048
IL-6 [pg/ml] & NK	0 .661	2 .489	0 .038
CD45 & CD8	0 .661	2 .489	0 .038
CD8 & CD3	0 .661	2 .489	0 .038
NK & IL-6 [pg/ml]	0 .661	2 .489	0 .038
IL-6 [pg/ml] & PVR dyne/s/cm-5]	0 .667	2 .366	0 .050
IL-2 [pg/ml] & Mean pressure in the right ventricle [mmHg]	0 .669	2 .384	0 .049
IL-4 [pg/ml] & Mean pressure in the right ventricle [mmHg]	0 .669	2 .384	0 .049
IL-10 [pg/ml] & mPAP [mmHg]	0 .669	2 .384	0 .049
BMI & CO [L/min.]	0 .683	2 .476	0 .042

IL-6 [pg/ml] & CD45	0 .697	2 .749	0 .025
CD3 & IL-6 [pg/ml]	0 .697	2 .749	0 .025
IL-2 [pg/ml] & Lym [10^3/mm^3]	0 .729	3 .017	0 .017
IL-4 [pg/ml] & Lym [10^3/mm^3]	0 .729	3 .017	0 .017
IFN-γ [pg/ml] & BMI	0 .791	3 .663	0 .006
CI [L/min/m2] & CO [L/min.]	0 .867	4 .596	0 .002
mPAP [mmHg] & RVSP [mmHg]	0 .870	4 .675	0 .002
CD4 & CD4/CD8	0 .903	5 .946	0 .000
mPAP [mmHg] & PASP [mmHg]	0 .929	6 .635	0 .000
PASP [mmHg] & RVSP [mmHg]	0 .967	9 .989	0 .000

Supplementary materials Table S5 Sperman rank correlation analysis for patients with IPAH

Parameters	R	t(N-2)	p-value
PVR dyne/s/cm-5] & CO [L/min.]	-0 .649	-4 .096	0 .000
PVR dyne/s/cm-5] & CI [L/min/m2]	-0 .592	-3 .522	0 .002
Wiek & 6MWT [m]	-0 .584	-3 .452	0 .002
CI [L/min/m2] & IL-10 [pg/ml]	-0 .489	-2 .685	0 .013
IL-4 [pg/ml] & IFN-γ [pg/ml]	-0 .485	-2 .657	0 .014
IL-4 [pg/ml] & IFN-γ [pg/ml]	-0 .485	-2 .657	0 .014
Wiek & IL-2 [pg/ml]	-0 .469	-2 .549	0 .018
IL-6 [pg/ml] & CD19	-0 .466	-2 .527	0 .019
Mean pressure in the right ventricle [mmHg] & IL-2 [pg/ml]	-0 .462	-2 .498	0 .020
CO [L/min.] & IL-2 [pg/ml]	-0 .432	-2 .295	0 .031
CO [L/min.] & IL-10 [pg/ml]	-0 .429	-2 .280	0 .032
Wiek & mPAP [mmHg]	-0 .426	-2 .260	0 .034
Wiek & PASP [mmHg]	-0 .416	-2 .193	0 .039
NT-proBNP [pg/ml] & IL-10 [pg/ml]	-0 .402	-2 .106	0 .046
PVR dyne/s/cm-5] & IL-6 [pg/ml]	0 .406	2 .131	0 .044
CD45 & CD4/CD8	0 .435	2 .319	0 .030
CD3 & CD4/CD8	0 .435	2 .319	0 .030
CO [L/min.] & IFN-γ [pg/ml]	0 .438	2 .338	0 .028
PVR dyne/s/cm-5] & IL-2 [pg/ml]	0 .444	2 .375	0 .026
IL-10 [pg/ml] & CD4/CD8	0 .444	2 .375	0 .026
CD8 & CD19	0 .465	2 .516	0 .019
Mean pressure in the right ventricle [mmHg] & mPAP [mmHg]	0 .470	2 .555	0 .018
IL-10 [pg/ml] & CD4	0 .475	2 .591	0 .016
CD4 & CD8	0 .477	2 .602	0 .016
6MWT [m] & RVSP [mmHg]	0 .518	2 .901	0 .008
mPAP [mmHg] & IL-6 [pg/ml]	0 .539	3 .066	0 .005
6MWT [m] & PASP [mmHg]	0 .568	3 .309	0 .003
CI [L/min/m2] & IFN-γ [pg/ml]	0 .581	3 .423	0 .002
CD45 & CD19	0 .613	3 .722	0 .001
CD19 & CD3	0 .613	3 .722	0 .001
CD4/CD8 & CD19	0 .693	4 .611	0 .000

**CD4 & CD19**	0 .703	4 .742	0 .000
**PVR dyne/s/cm-5] & mPAP [mmHg]**	0 .724	5 .036	0 .000
**CD45 & CD4**	0 .812	6 .680	0 .000
**CD3 & CD4**	0 .812	6 .680	0 .000
**CD4 & CD4/CD8**	0 .816	6 .774	0 .000
**CD45 & CD8**	0 .838	7 .379	0 .000
**CD3 & CD8**	0 .838	7 .379	0 .000
**CI [L/min/m2] & CO [L/min.]**	0 .869	8 .432	0 .000
**PASP [mmHg] & RVSP [mmHg]**	0 .982	25 .057	0 .000
